# Supplementary material for: Anatomical dissociation of intracerebral signals for reward and punishment prediction errors in humans
Source: Nat Commun. 2021 Jun 7;12:3344. doi: 10.1038/s41467-021-23704-w (PMC8184756; doi:10.1038/s41467-021-23704-w)
Supplement: Supplementary file 1 — Supplementary Information [file 41467_2021_23704_MOESM1_ESM.pdf]

Supplementary information

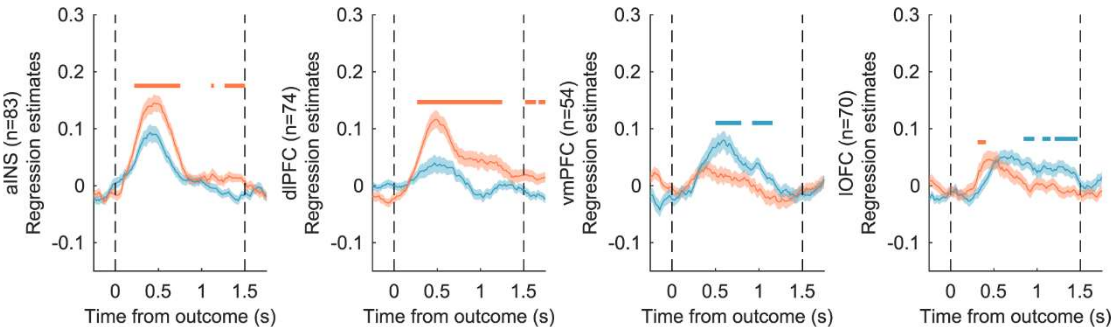

Supplementary Figure 1. Dissociation of reward PE (R-Qr) and punishment PE (P-Qp) signals using a single bandpass filter to estimate BGA. Time course of regression estimates obtained from linear fit of BGA with PE modeled separately for the reward (blue) and punishment (red) conditions (PPE: punishment prediction error; RPE: reward prediction error). Horizontal bold lines indicate significant difference between conditions (blue: RPE>PPE; red: PPE>RPE; $p_c<0.05$). Shaded areas represent inter-sites SEM. n indicates the number of recording sites in each ROI.

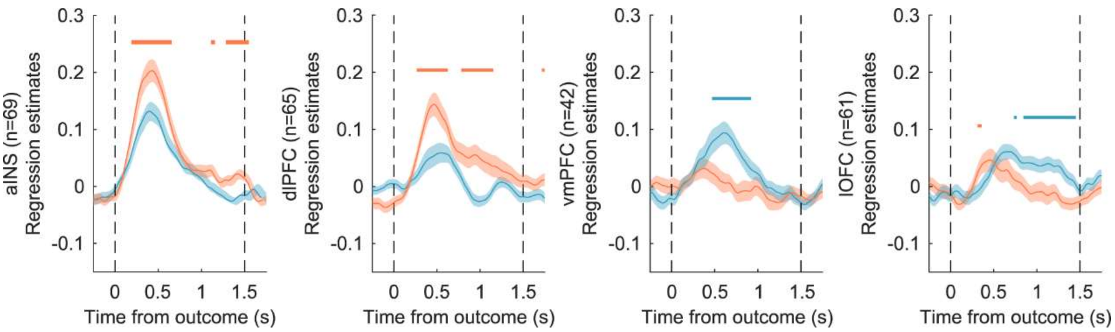

Supplementary Figure 2. Dissociation of reward PE (R-Qr) and punishment PE (P-Qp) signals after excluding channels and trials contaminated with pathological activity and/or artifacts. Time course of regression estimates obtained from linear fit of BGA with PE modeled separately for the reward (blue) and punishment (red) conditions (PPE: punishment prediction error; RPE: reward prediction error). Horizontal bold lines indicate significant difference between conditions (blue: RPE>PPE; red: PPE>RPE; $p_c<0.05$). Shaded areas represent inter-sites SEM.

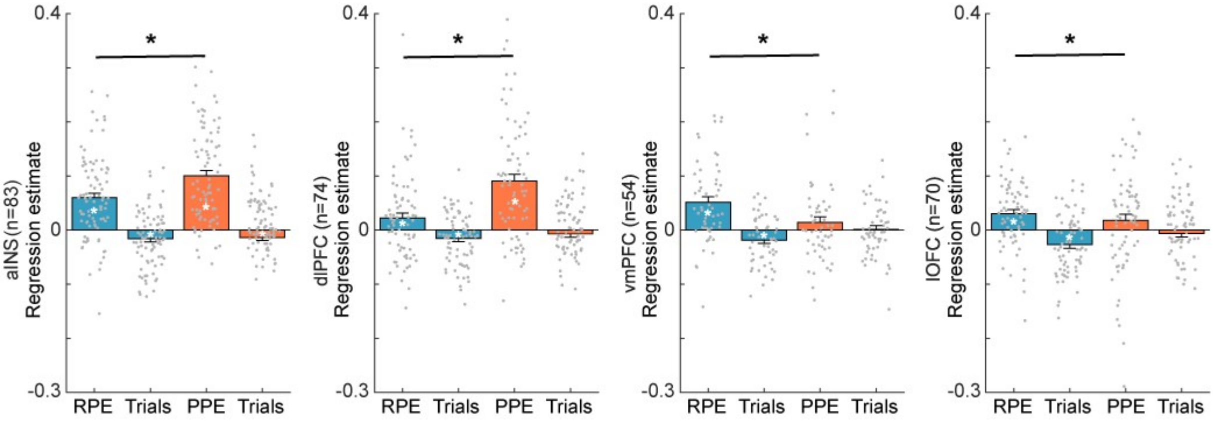

Supplementary Figure 3. Dissociation of reward PE (R-Qr) and punishment PE (P-Qp) signals after controlling for trial number. Averaged regression estimates obtained from a linear from linear fit of BGA with PE and trial index modeled separately for the reward (RPE and TRIALS: blue) or punishment (PPE and TRIALS: red) condition. Regression estimates were averaged over a 0.25-.1 s time window. Stars indicate significance (* p<0.05, white: one-sample, two-tailed Student's t-test; black: paired-samples, two-tailed Student's t-test). n indicates the number of recording sites in each ROI. Error-bars correspond to inter-sites SEM and dots correspond to individual recording sites.

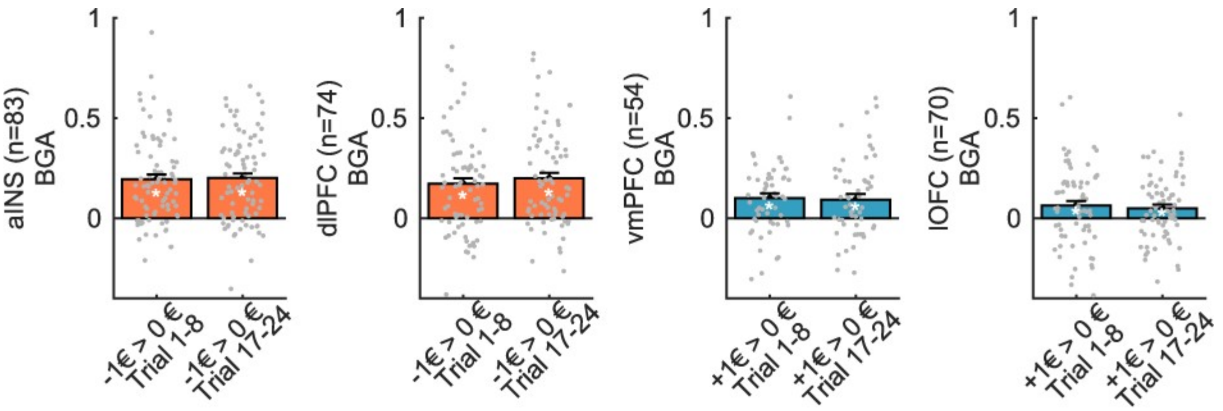

Supplementary Figure 4. Stability of contrasts between punishment (reward) and non-punishment (non-reward) outcomes in aINS and dlPFC (vmPFC and lOFC) over the course of learning. Average broadband gamma responses (over the 0.25-1 s time window) to relative punishments (i.e., the average of the difference of BGA between -1 € and 0€ outcomes) or to relative rewards (+1 € vs. 0€) split as a function of learning phase (early vs late trials). Early trials corresponded to the first 8 trials; Late trials corresponded to the last 8 trials. Stars indicate significance (* p<0.05, one-sample, two-tailed Student's t-test). n indicates the number of recording sites in each ROI. Error-bars correspond to inter-sites SEM and dots correspond to individual recording sites.

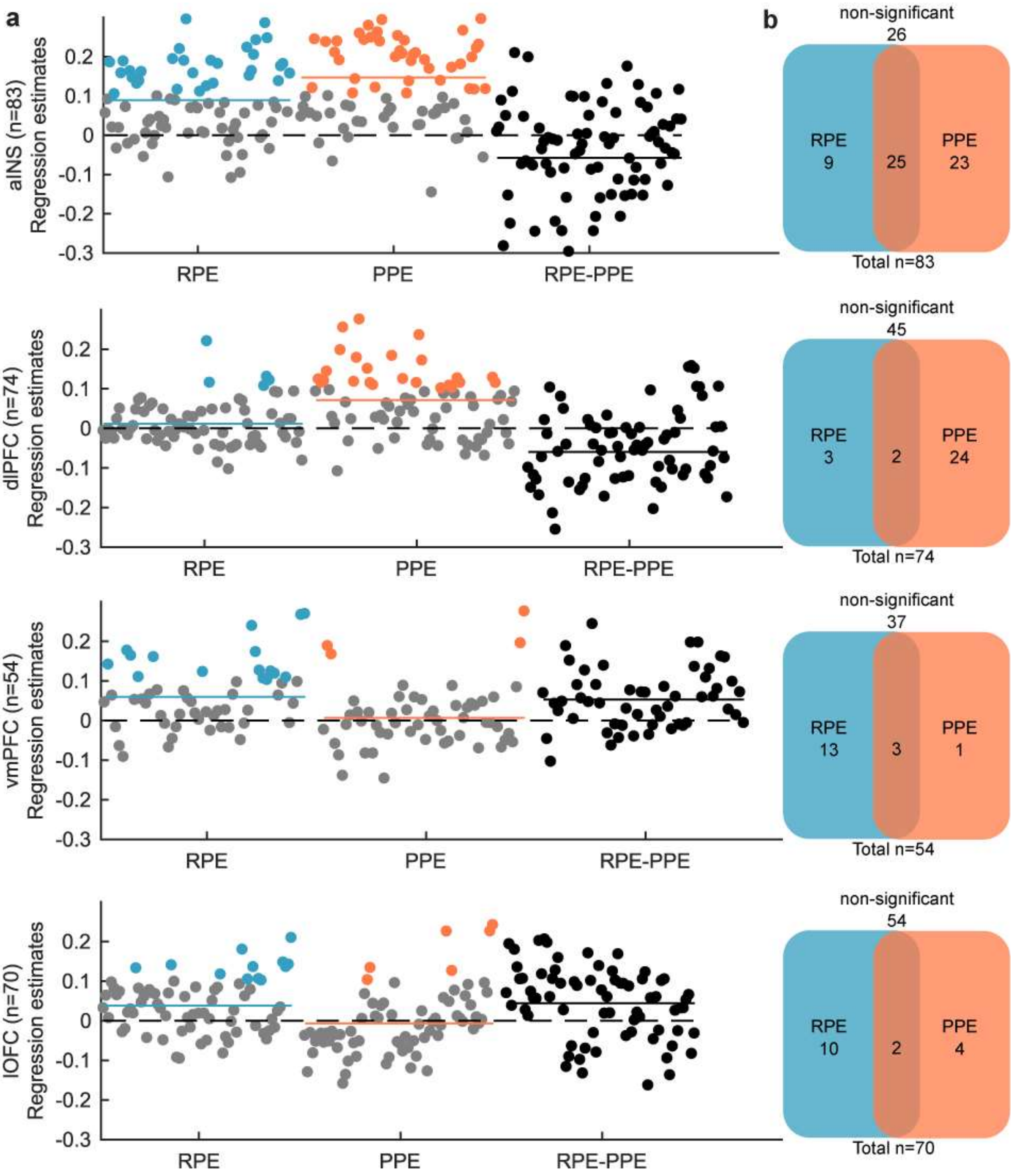

Supplementary Figure 5. Dissociation of reward and punishment PE signals across recording sites. a. Average regression estimates obtained from linear fit of BGA with PE modeled separately for the reward and punishment conditions (PPE: punishment prediction error; RPE: reward prediction error) for all recording sites within each region of interest (estimates were averaged within the cluster in which the RPE vs. PPE contrast was significantly). Horizontal lines indicate average across sites (blue: RPE; red: PPE; black: RPE-PPE). n indicates the number of recording sites in each ROI. **b**. Venn diagram indicating the number of recording sites encoding RPE and/or PPE (or nothing).

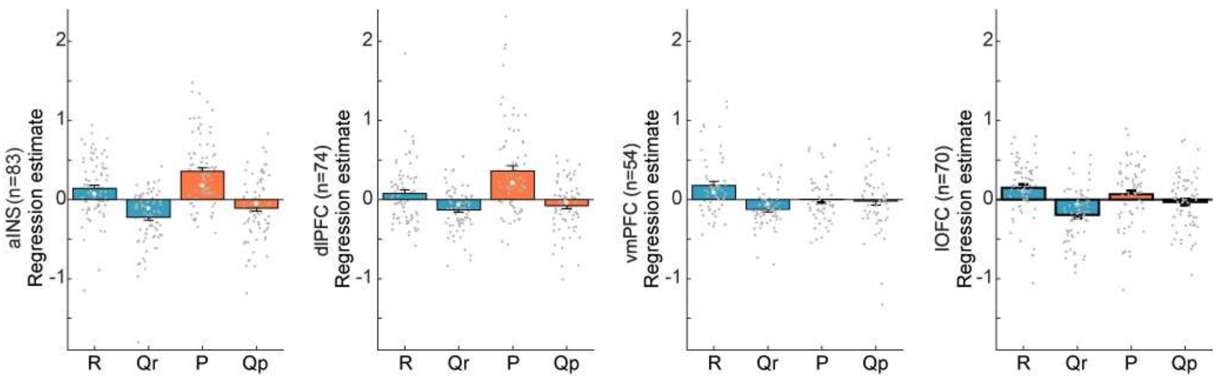

Supplementary Figure 6. Decomposed PE signals into outcome and expected value by domains (RPE: R and Qr; PPE: P and Qp) after using a fixation preceding stimulus onset to normalize BGA. Averaged regression estimates obtained from a linear from linear fit of BGA with PE components modeled separately for the reward (R or Qr: blue) or punishment (P or Qp: red) condition. Regression estimates were averaged over a 0.5-.75 s time window. Stars indicate significance (* $p<0.05$, one-sample, two-tailed Student's t-test). n indicates the number of recording sites in each ROI. Error-bars correspond to inter-sites SEM and dots correspond to individual recording sites.

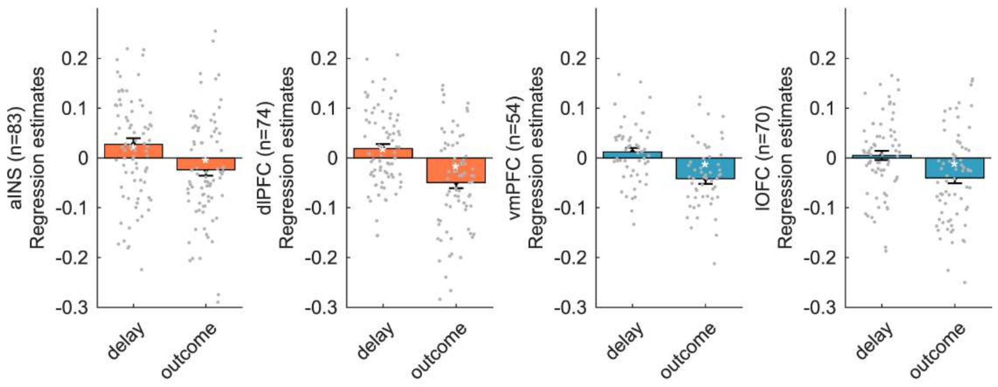

Supplementary Figure 7. Expectation signals during the delay vs. outcome phase. Average regression estimates obtained from linear fit of BGA with expectation modeled separately for the punishment condition (Qp: punishment expectation) or the reward condition (Qr: reward expectation). Time windows used to average regression estimates were time-locked to outcome onset: Delay time window corresponds to [-1.2 to -.2 s]; Outcome time window corresponds to [.25 to 1s]. Stars indicate significance (* $p<0.05$, one-sample, two-tailed Student's t-test). n indicates the number of recording sites in each ROI. Error-bars correspond to inter-sites SEM and dots correspond to individual recording sites.

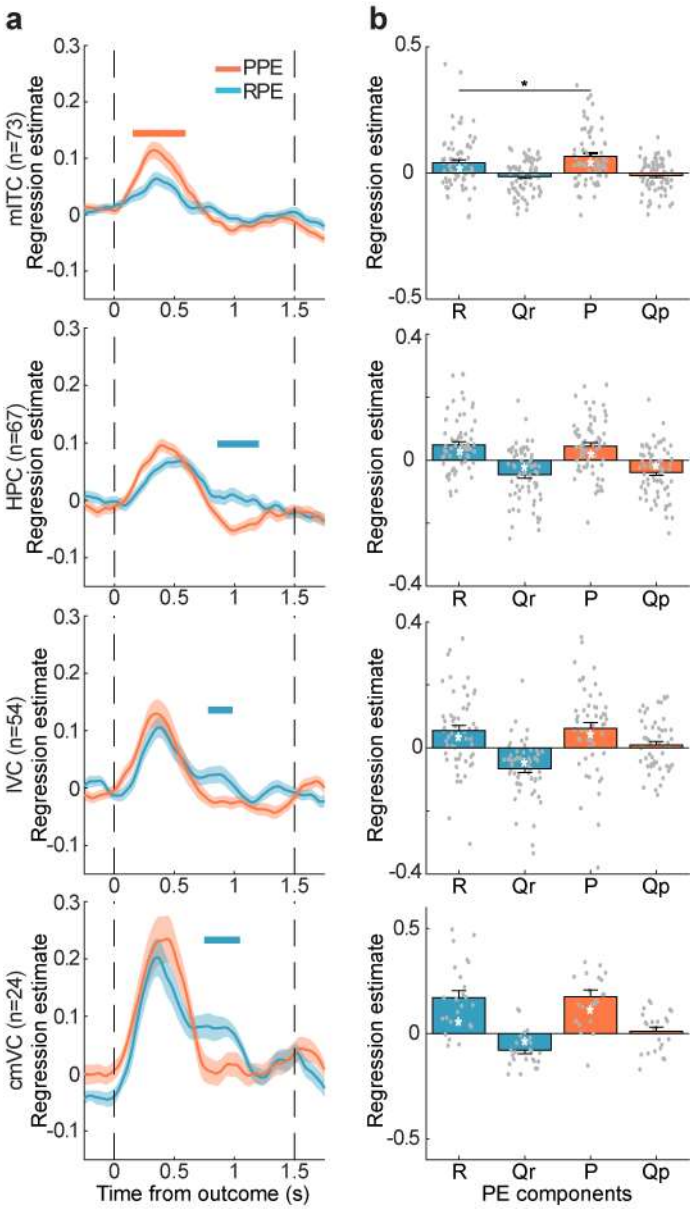

Supplementary Figure 8. Reward and punishment PE signals in supplementary PE regions. a. Time course of regression estimates obtained from linear fit of BGA with PE modeled separately for the reward and punishment conditions. Horizontal bold lines indicate significant difference between conditions (blue: RPE>PPE; red: PPE>RPE; $p_c<0.05$). Shaded areas represent inter-patient SEM. **b.** Regression estimates of broadband gamma power against prediction error components averaged over the 0.25-1 s post-outcome time window. lVC: lateral visual cortex; cmVC: caudal medial visual Cortex; mITC: medial inferior temporal cortex; HPC: hippocampus. n indicates the number of recording sites in each ROI. Error-bars correspond to inter-sites SEM and dots correspond to individual recording sites. Stars indicate significance (* p<0.05, one-sample, two-tailed Student's t-test).

Supplementary Table 1 : Demographical and clinical details for which written informed consent was obtained from all participants. Abbreviations used: orbitofrontal cortex: OFC; premotor cortex: PM; hippocampus: HPC; left: L; right: R; alprazolam: APZ; carbamazepine: CBZ; clobazam: CLB; gabapentin: GBP; lacosamide: LCM, lamotrigine: LMT; levetiracetam: LEV, nitrazepam: NZP; oxcarbazepine: OXC; perampanel: PER; topiramate: TPM; sodium valproate: VPA; zonisamide: ZON.

Patient	Sex	Age	Hand laterality	Number of iEEG sites	Epileptic focus	Epilepsy onset (age)	Antiepileptic drugs
1	F	29	R	98	L frontopolar	19	LCM + LMT
2	F	42	R	104	L temporal	13	CLB + LEV + ZON
3	F	20	R	98	L temporal	11	CLB + LCM + LMT
4	M	48	L	98	R temporal	12	OXC
5	M	37	R	100	L OFC	4	LCM + NZP
6	F	13	R	80	R PM	10	CBZ + VPA
7	F	34	R	80	L HPC + insular	5	LCM + LEV
8	M	32	R	96	Bilateral temporal	21	LTG + PER
9	F	15	R	91	R temporal + insular	7	CLB + LEV
10	M	28	R	155	R fronto-temporal	11	CBZ + LCM
11	M	20	R	161	G frontal + temporal (2)	14	LCM + LMT + ZON
12	M	41	R	135	R temporal	32	CLB + GBP
13	F	15	R	99	L temporo-occipital	12	CLB + LCM + LMT
14	M	40	R	110	R temporal	5	CBZ + LCM
15	F	47	R	101	R insular + L frontal	7	LCM + TPM
16	F	34	R	90	R OFC + temporal	28	CBZ + LCM + LMT
17	M	57	R	107	L temporal	37	CBZ + LMT + PER
18	M	41	L	45	L temporal		CBZ + CLB
19	M	29	R	99	R OFC	25	LCM + LMT
20	F	48	R	136	R temporal	19	LCM + LEV + LMT

Supplementary Table 2: Significance of prediction-error signals for the entire dataset.

Areas (MarsAtlas labels) are ordered according to the t-value obtained by testing against zero (across sites within each parcel) the regression estimates of BGA against prediction errors (in the 0-1000 ms post-outcome time window). Bold p-values indicate significance after correction for multiple comparisons across regions (n=39 areas; $\alpha_{corrected}=1.3\times10^{-3}$ after Bonferroni correction; one-sample, two-tailed Student's t-tests). Grey: areas including either less than 9 recorded sites or a proportion of significant contact inferior to 0.2. lVC: lateral visual cortex; cmVC: caudal medial visual Cortex; mITC: medial inferior temporal cortex; HPC: hippocampus. pINS: posterior Insula.

Parcellation label	Number of iEEG sites	Number of patients	t-value	p-value	Number of significant patients	Proportion of significant contacts
**aINS**	83	13	11,89	**$1,59\times10^{-19}$**	11	0,48
**dlPFC**	74	9	7,72	**$4,69\times10^{-11}$**	8	0,38
**cmVC**	24	7	6,65	**$8,81\times10^{-7}$**	5	0,54
**HPC**	67	12	6,49	**$1,31\times10^{-8}$**	8	0,22
**mITC**	73	13	5,02	**$3,60\times10^{-6}$**	6	0,27
**lVC**	54	8	4,95	**$7,97\times10^{-6}$**	8	0,39
**lOFC**	70	10	4,26	**$6,45\times10^{-5}$**	7	0,21
**vmPFC**	54	11	3,98	**$2,10\times10^{-4}$**	4	0,22
Sdm	4	2	5,11	0,01	1	0,20
SPC	9	4	4,40	$2,28\times10^{-3}$	3	0,56
ACC	18	7	4,21	**$5,85\times10^{-4}$**	2	0,17
MTCr	111	11	3,20	$1,82\times10^{-3}$	2	0,05
Mv	27	6	3,19	0,00	3	0,19
SPCm	6	2	3,15	0,03	2	0,33
PCC	19	8	3,11	$6,02\times10^{-3}$	3	0,21
pINS	66	16	3,08	0,00	5	0,08
ITCr	36	8	3,03	$4,60\times10^{-3}$	2	0,33
IPCv	63	10	2,92	$4,93\times10^{-3}$	2	0,08
IPCd	19	5	2,79	0,01	1	0,16
PFrd	14	5	2,79	0,02	1	0,07
PMrv	31	7	2,74	0,01	3	0,13
Mdl	25	5	2,70	0,01	2	0,32
PFrvl	15	6	2,62	0,02	2	0,40
PFcdm	13	5	2,59	0,02	2	0,31
MTCc	172	13	2,41	0,02	5	0,06
Sv	20	4	2,08	0,05	1	0,10
PMdm	5	2	1,59	0,19	1	0,20
MCC	6	4	1,39	0,22	0	0,00
VCs	18	6	1,33	0,20	3	0,22
PFcdl	25	9	1,03	0,31	1	0,04
STCc	105	15	1,02	0,31	1	0,01
Sdl	15	4	0,60	0,56	1	0,13
PCm	12	5	-0,03	0,98	1	0,08
PMdl	15	5	-0,38	0,71	1	0,07
PFrm	14	8	-0,40	0,70	0	0,00
Mdm	2	1	-0,79	0,57	0	0,00
VCrm	8	3	-1,40	0,20	0	0,00
STCr	77	12	-1,72	0,09	3	0,04
